# Supplementary material for: Caspase 9-induced apoptosis enables efficient fetal cell ablation and disease modeling
Source: Nat Commun. 2025 Mar 15;16:2572. doi: 10.1038/s41467-025-57795-6 (PMC11910536; doi:10.1038/s41467-025-57795-6)
Supplement: Supplementary file 1 — Supplementary Information [file 41467_2025_57795_MOESM1_ESM.pdf]

Caspase 9-induced apoptosis enables efficient fetal cell ablation and disease modeling

Kenji Matsui[1], Masahito Watanabe[2†], Shutaro Yamamoto[1,3], Shiho Kawagoe[1], Takumi Ikeda[1], Hinari Ohashi[1], Takafumi Kuroda[1], Nagisa Koda[1], Keita Morimoto[1], Yoshitaka Kinoshita[1,4], Yuka Inage[1,5], Yatsumu Saito[1], Shohei Fukunaga[1], Toshinari Fujimoto[1], Susumu Tajiri[1], Kei Matsumoto[1], Eiji Kobayashi[6], Takashi Yokoo[1*] and Shuichiro Yamanaka[1,7*]

[1]Division of Nephrology and Hypertension, Department of Internal Medicine, The Jikei University School of Medicine, Nishi-Shimbashi 3-25-8, Minato-ku, 105-8461 Tokyo, Japan.
[2]Meiji University International Institute for Bio-Resource Research, Higashimita 1-1-1, Tama-ku, Kawasaki, 214-8571 Kanagawa, Japan.
[3]Department of Urology, The Jikei University School of Medicine, Nishi-Shimbashi 3-25-8, Minato-ku, 105-8461 Tokyo, Japan
[4]Department of Urology, Graduate School of Medicine, The University of Tokyo, Hongo 7-3-1, Bunkyo-ku, 113-0033 Tokyo, Japan.
[5]Department of Pediatrics, The Jikei University School of Medicine, Nishi-Shimbashi 3-25-8, Minato-ku, 105-8461 Tokyo, Japan
[6]Department of Kidney Regenerative Medicine, The Jikei University School of Medicine, Nishi-Shimbashi 3-25-8, Minato-ku, 105-8461 Tokyo, Japan
[7]Kidney Applied Regenerative Medicine, Project Research Units, The Jikei University School of Medicine, Nishi-Shimbashi 3-25-8, Minato-ku, 105-8461 Tokyo, Japan.
†Present affiliation: PorMedTec Co., Ltd., 2-3227 Mita, Tama-ku, Kawasaki, 214-0034 Kanagawa, Japan
*Corresponding authors, shu.yamanaka@jikei.ac.jp; tyokoo@jikei.ac.jp

Table of Contents

- Supplementary Fig. 1 | Validation of the iC9 vector and diagnosis of Six2-iC9 mice.
- Supplementary Fig. 2 | The effect of CID on wild-type fetuses.
- Supplementary Fig. 3 | Neonatal wild-type mice kidneys.
- Supplementary Fig. 4 | Analysis of a chronic kidney disease model by CID administration during a fetal period.
- Supplementary Fig. 5 | NPC ablation in homozygous neonatal kidneys.
- Supplementary Fig. 6 | Fetal mice kidney culture on the air–liquid interface.
- Supplementary Fig. 7 | Slow apoptosis induction in Six2-DTA mice.
- Supplementary Fig. 8 | Ineffective NPC ablation with CID in fetal heterozygous kidneys.
- Supplementary Fig. 9 | Evaluation of protein expression levels of using capillary Western immunoassay.
- Supplementary Fig. 10 | Cell ablation induction in dissociated and re-aggregated fetal Six2-iC9$^{+/+}$ kidney cell spheres.
- Supplementary Fig. 11 | NPC ablation in heterozygous neonatal kidneys by co-administration of XIAP inhibitor and CID.
- Supplementary Fig. 12 | Replacement and maturation of nephron progenitor cells using fetal Six2-iC9 kidneys as a scaffold.
- Supplementary Fig. 13 | Purity and differentiation capacity of induced human NPCs.
- Supplementary Fig. 14 | Comparison of the size of regenerated nephrons between humans and mice.
- Supplementary Fig. 15 | A schematic illustrating this study.
- Supplementary Fig. 16 | An uncropped scan of all blots in Supplementary Fig. 1c.

- Supplementary Table 1 | The primer sequences for genomic PCR.
- Supplementary Table 2 | List of primary antibodies for immunostaining.
- Supplementary Table 3 | List of primary antibodies for capillary Western immunoassay.
- Supplementary Table 4 | List of TaqMan Gene Expression Assays for RT-qPCR.

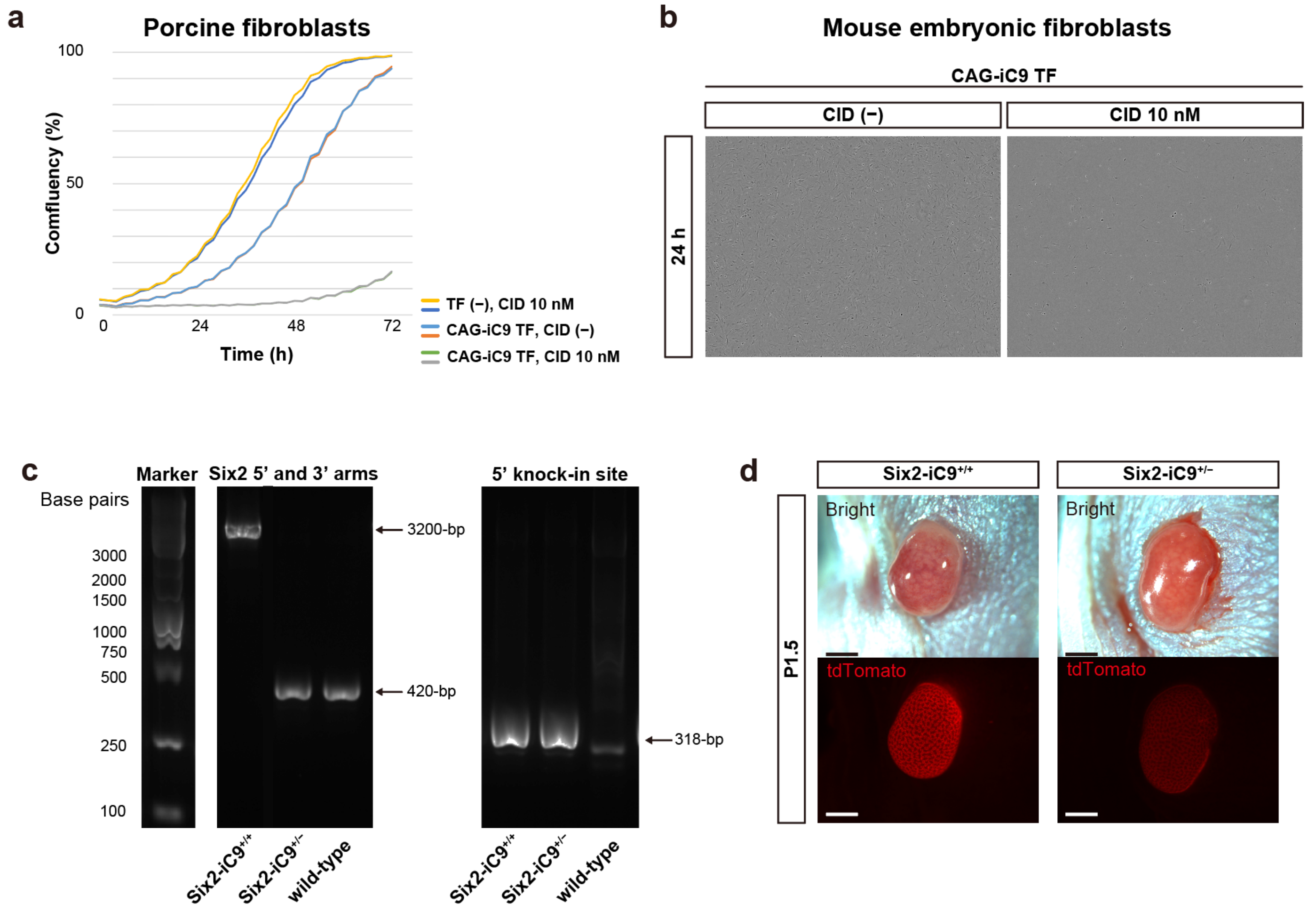

Supplementary Fig. 1 | Validation of the iC9 vector and diagnosis of Six2-iC9 mice.
a Confluency of porcine fibroblasts cultured over time after transfection with the CAG-iC9 vector. Cell proliferation is inhibited when 10 nM CID was added to the transfected fibroblasts. **b** Phase-contrast images of mouse embryonic fibroblasts cultured for 24 hours after transfection with the CAG-iC9 vector. **c** Representative data of genomic PCR to differentiate Six2-iC9$^{+/+}$ (homozygous), Six2-iC9$^{+/-}$ (heterozygous), and wild-type mice. **d** Fluorescence stereomicroscopic images of exposed left kidneys of Six2-iC9$^{+/+}$ and Six2-iC9$^{+/-}$ neonates on postnatal day 1.5 (P1.5) under the same imaging conditions, showing tdTomato^{+} NPCs. Scale bars, 1 mm. CID, chemical inducer of dimerization; TF, transfection.

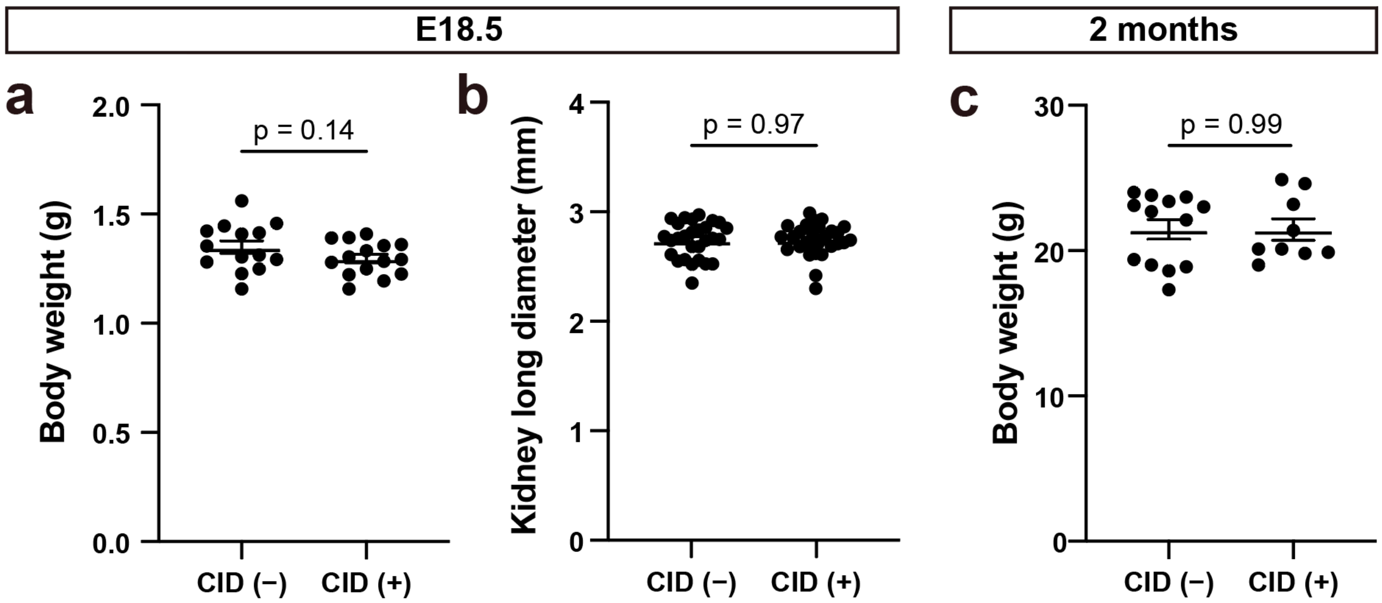

Supplementary Fig. 2 | The effect of CID on wild-type fetuses.

a Body weight of E18.5 wild-type fetuses extracted by cesarean section without CID administration (CID (−)) and those after intraperitoneal administration of CID on E13.5 (CID (+)). **b** Kidney long diameter of fetuses of the CID (−) group and the CID (+) group. **c** Body weight at 2 months of age of the CID (−) group and the CID (+) group. For (**a**), 14 fetuses derived from 2 mothers and 15 fetuses derived from 2 mothers were analyzed for the CID (−) and CID (+) group, respectively. For (**b**), 28 and 30 kidneys from these fetuses were analyzed in the CID (−) and CID (+) group, respectively. For (**c**), 13 fetuses derived from 2 mothers and 9 fetuses derived from 2 mothers were analyzed for the CID (−) and CID (+) group, respectively. The data are presented as mean ± SEM. Statistical analysis was performed using a two-tailed unpaired *t*-test. Source data are provided as a Source Data file. CID, chemical inducer of dimerization.

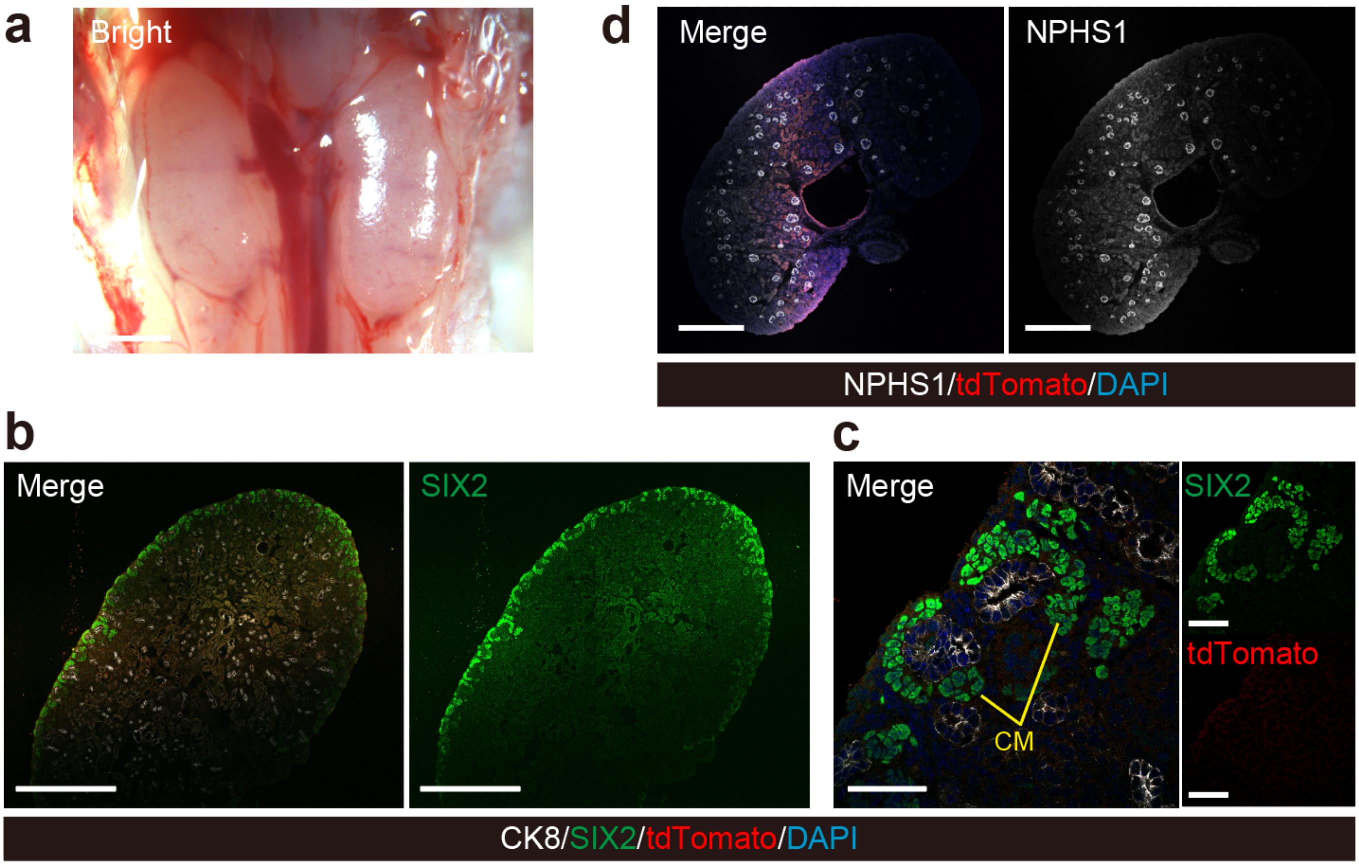

Supplementary Fig. 3 | Neonatal wild-type mice kidneys.

a A stereomicroscopic image of P0.5 neonatal wild-type kidneys. Scale bar, 1 mm. **b** Immunostaining images of (**a**) for SIX2^{+}/tdTomato^{-} NPCs surrounding CK8^{+} ureteric buds. Scale bars, 500 μm. **c** Magnified images of (**b**). Scale bars, 50 μm. **d** Immunostaining images of (**a**) for NPHS1^{+} mature glomeruli. Scale bars, 500 μm. CK8, cytokeratin 8; CM, cap mesenchyme.

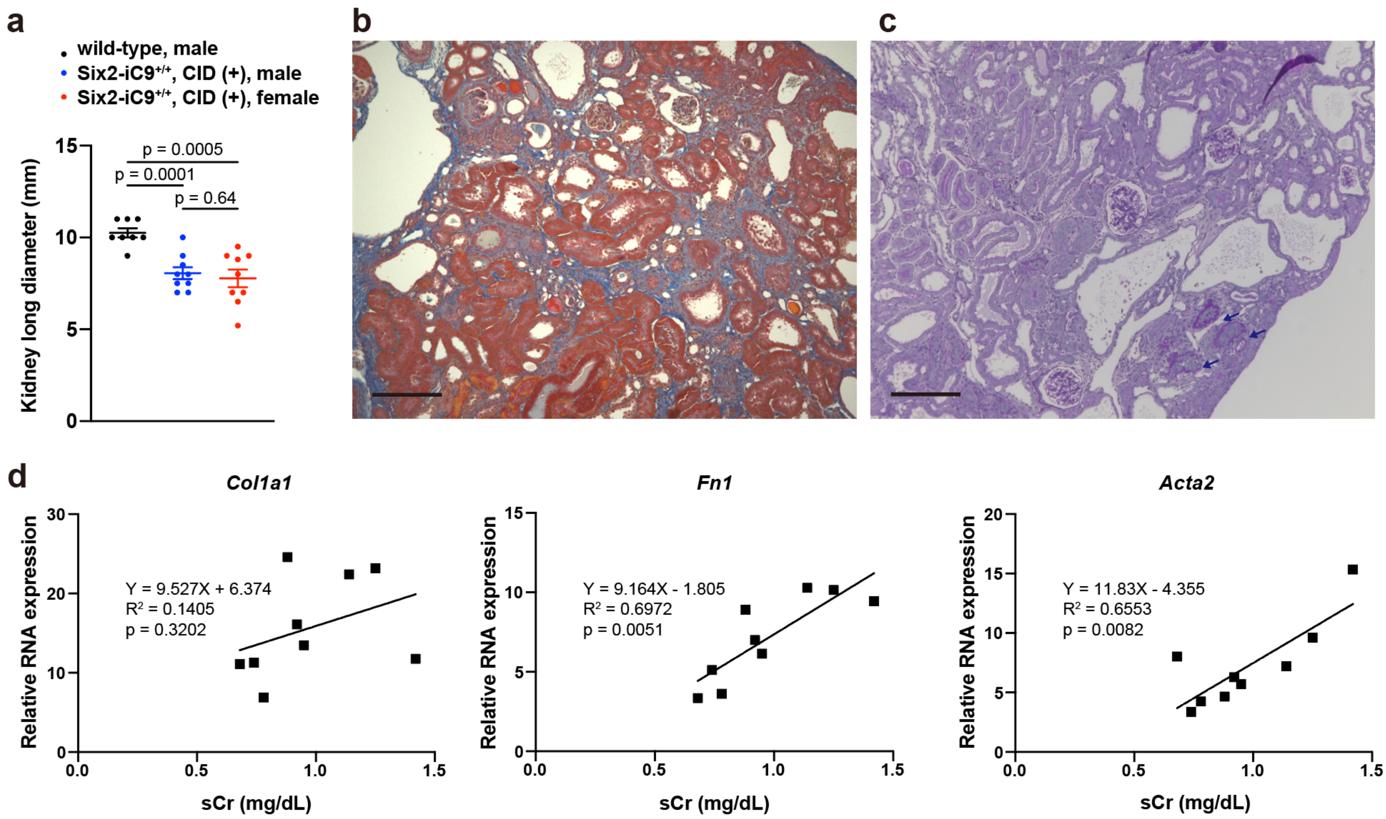

Supplementary Fig. 4 | Analysis of a chronic kidney disease model by CID administration during a fetal period. **a** Long diameter of right kidneys of male wild-type, male and female Six2-iC9$^{+/+}$ administered CID on E13.5 at 2 months of age. The data were analyzed using 8 male wild-type, 9 male Six2-iC9$^{+/+}$, and 9 female Six2-iC9$^{+/+}$ individuals and are presented as mean ± SEM. **b, c** Masson's trichrome staining (**b**) and periodic acid-schiff staining (**c**) of kidneys at 2 months of age of male Six2-iC9$^{+/+}$ mice administered CID on E13.5. Blue arrows in (**c**) indicate atrophic tubules. Scale bars, 200 µm. **d** Correlation of relative RNA expression of *Col1a1*, *Fn1*, and *Acta2* with sCr levels in male Six2-iC9$^{+/+}$ administered CID on E13.5 at 2 months of age. Linear regression analysis was performed using 9 male Six2-iC9$^{+/+}$ individuals and is shown with equations, R^2 values, and p-values provided in each plot. Source data are provided as a Source Data file. *Acta2*, actin alpha 2, smooth muscle; *Col1a1*, collagen type I alpha 1 chain; *Fn1*, fibronectin 1; sCr, serum creatinine.

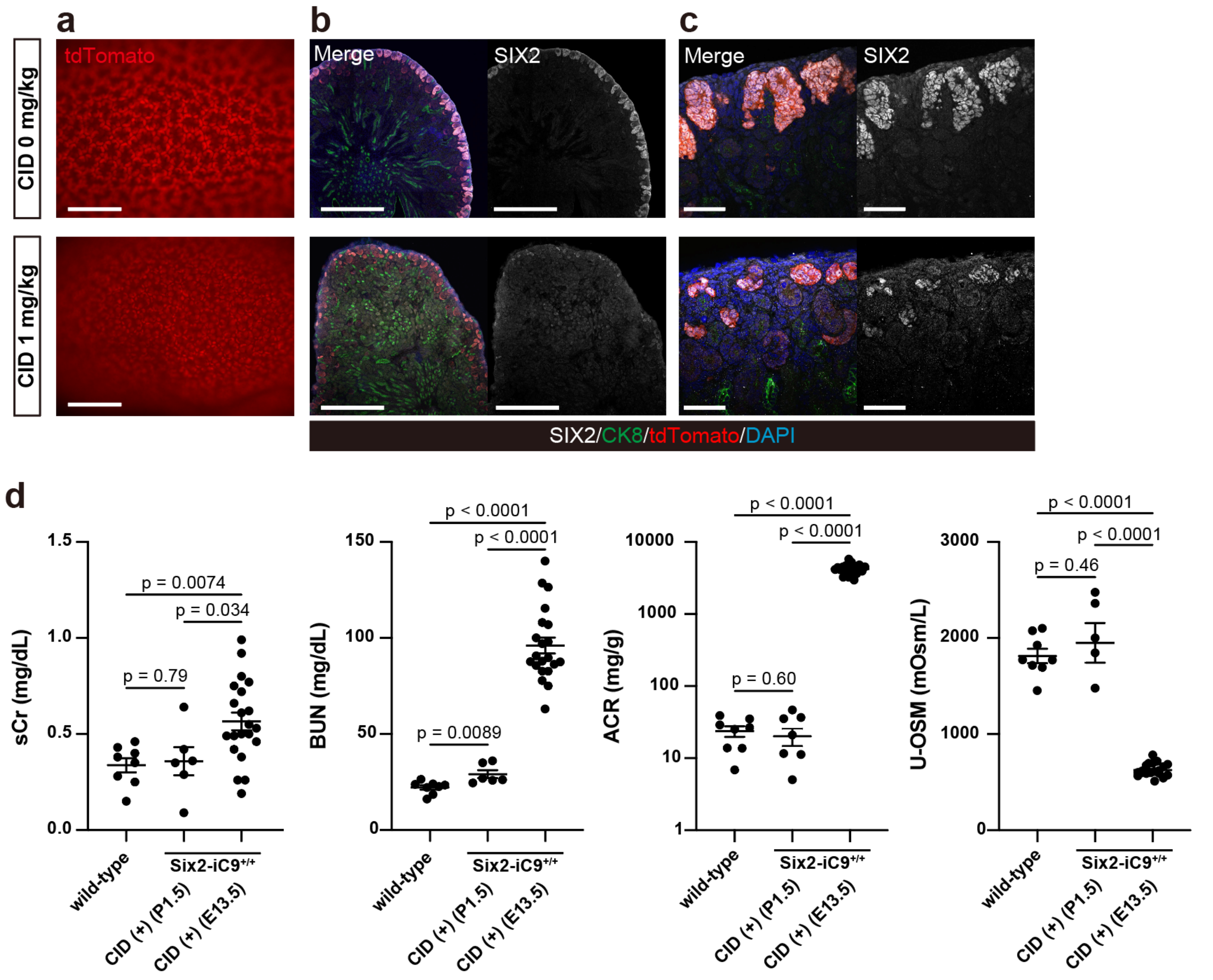

Supplementary Fig. 5 | NPC ablation in homozygous neonatal kidneys.

a Fluorescence stereomicroscopic images of P2.5 neonatal Six2-iC9$^{+/+}$ kidneys without drug administration or 1 day after subcutaneous administration of 1 mg/kg CID on P1.5. Scale bars, 500 µm. **b** Frozen section immunostaining images of (**a**). Scale bars, 500 µm. **c** Magnified images of (**b**). Scale bars, 50 µm. **d** Serum creatinine (sCr), blood urea nitrogen (BUN), urinary albumin/creatinine ratio (ACR), and urinary osmolality (U-OSM) in Six2-iC9$^{+/+}$ mice of both sexes treated with CID on P1.5 or E13.5 at 1 month of age, compared with those of male wild-type mice. The data were analyzed using 9 wild-type mice, 6 Six2-iC9$^{+/+}$ mice treated on P1.5, and 21 Six2-iC9$^{+/+}$ mice treated on E13.5, and are presented as mean ± SEM. Source data are provided as a Source Data file. CID, chemical inducer of dimerization; CK8, cytokeratin 8.

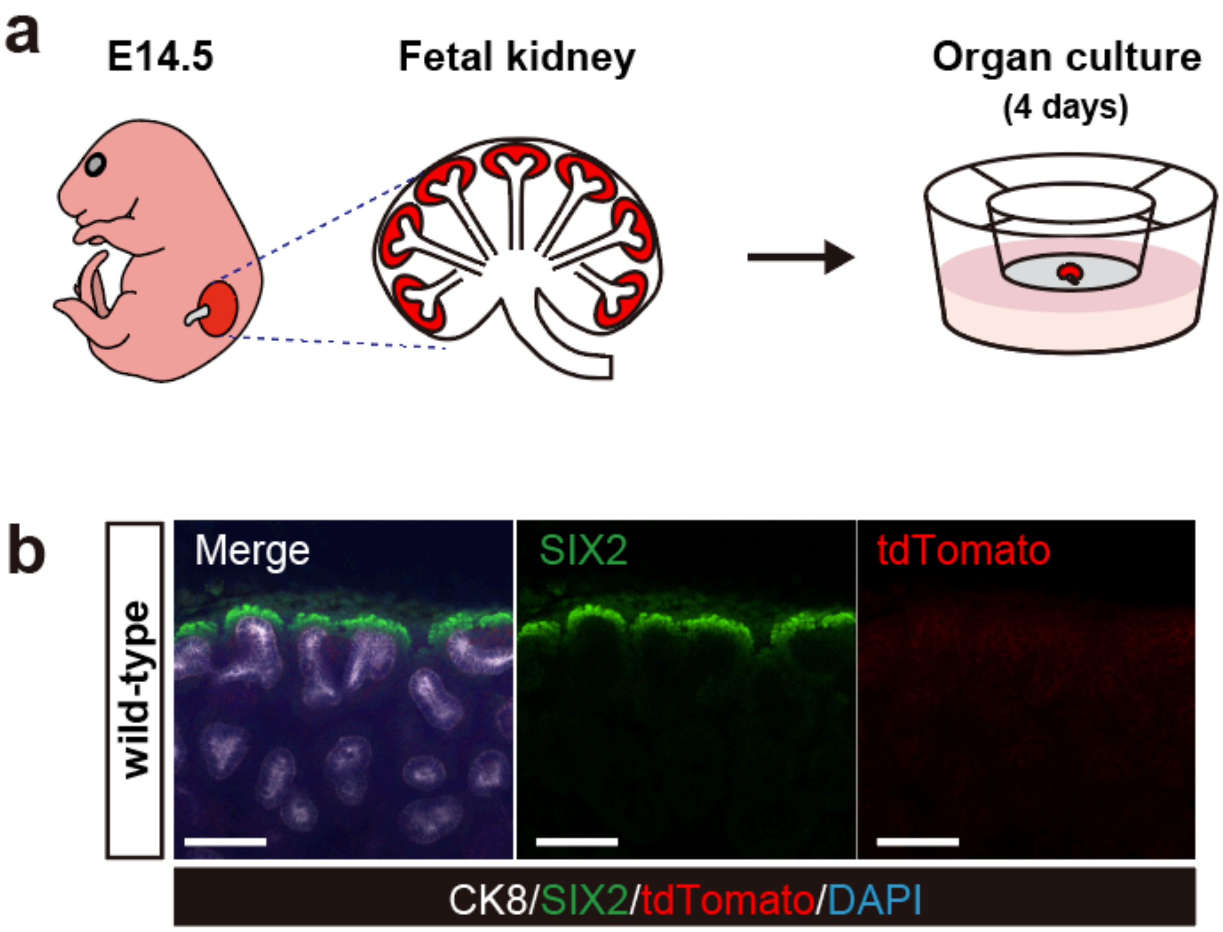

Supplementary Fig. 6 | Fetal mice kidney culture on the air–liquid interface.
a A schematic illustrating the culture of embryonic day 14.5 (E14.5) fetal kidneys on the air–liquid interface. **b** Whole-mount immunostaining images of fetal wild-type kidneys cultured for 4 days. Scale bars, 100 μm. CK8, cytokeratin 8.

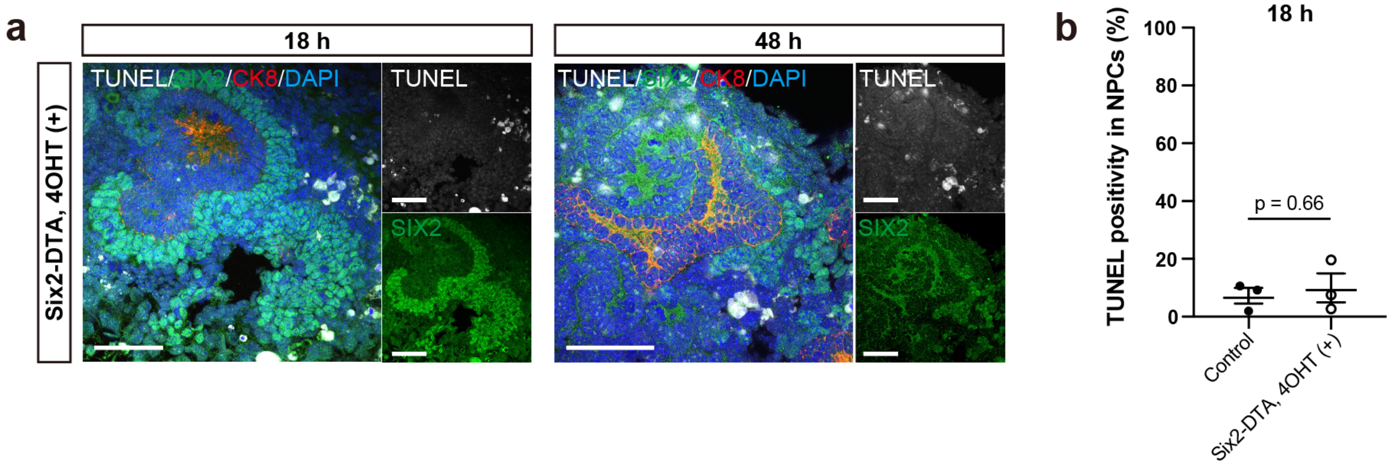

Supplementary Fig. 7 | Slow apoptosis induction in Six2-DTA mice.

a Frozen section images of TUNEL staining along with immunostaining for $SIX2^{+}$ NPCs and $CK8^{+}$ ureteric buds of fetal Six2-DTA kidneys harvested 18 and 48 hours after the start of culture with 2 μg/mL 4OHT. Scale bars, 50 μm. **b** The percentage of NPCs under apoptosis ($TUNEL^{+}$/$SIX2^{+}$/$DAPI^{+}$) in NPCs ($SIX2^{+}$/$DAPI^{+}$) in fetal Six2-DTA kidneys at 18 hours compared to controls (fetal Six2-iC9$^{+/+}$ kidneys cultured without CID). Data were analyzed using 3 biologically independent samples and are presented as mean ± SEM. Statistical analysis was performed using a two-tailed unpaired *t*-test. Source data are provided as a Source Data file. 4OHT, 4-hydroxytamoxifen; CID, chemical inducer of dimerization; CK8, cytokeratin 8; TUNEL, terminal deoxynucleotidyl transferase dUTP nick end labelling.

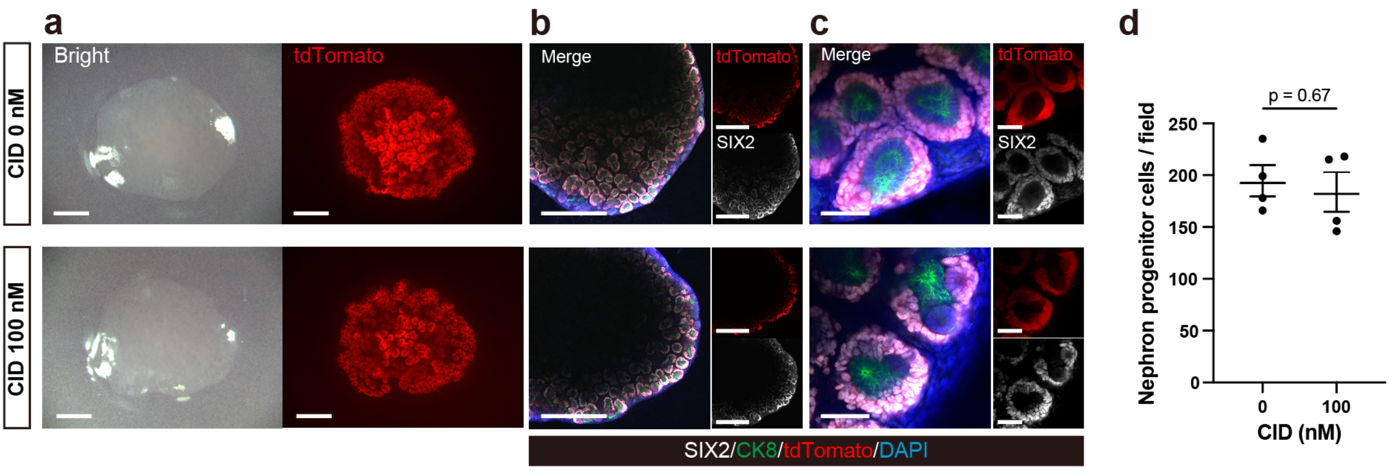

Supplementary Fig. 8 | Ineffective NPC ablation with CID in fetal heterozygous kidneys.
a Fluorescence stereomicroscopic images of fetal Six2-iC9$^{+/-}$ kidneys cultured without CID and with 100 nM CID for 4 days on the air–liquid interface, showing tdTomato^{+} NPCs. Scale bars, 500 μm. **b** Whole-mount immunostaining images of (**a**), showing SIX2^{+}/tdTomato^{+} NPCs surrounding CK8^{+} ureteric buds. Scale bars, 500 μm. **c** Magnified images of (**b**). Scale bars, 50 μm. **d** The number of NPCs per field after 4 days of culture with CID treatment. Data were analyzed using 4 biologically independent samples and are presented as mean ± SEM. Statistical analysis was performed using a two-tailed unpaired *t*-test. Source data are provided as a Source Data file. CID, chemical inducer of dimerization; CK8, cytokeratin 8.

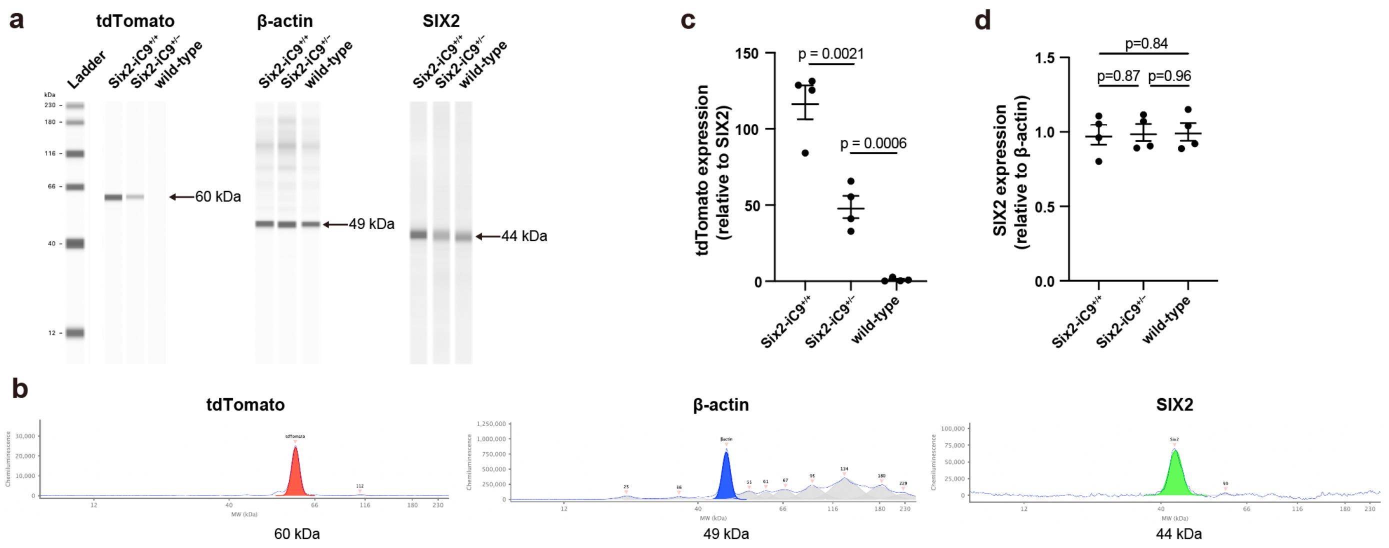

Supplementary Fig. 9 | Evaluation of protein expression levels of using capillary Western immunoassay.

a Lane view of Wes for tdTomato, β-actin, and SIX2. **b** Representative graph images of Wes for tdTomato, β-actin, and SIX2. **c** Relative expression levels of SIX2 compared to β-actin in kidneys of fetal Six2-iC9$^{+/+}$, Six2-iC9$^{+/-}$, and wild type mice. **d** Relative expression levels of tdTomato compared to SIX2 in kidneys of fetal Six2-iC9$^{+/+}$, Six2-iC9$^{+/-}$, and wild type mice. Data were analyzed using 4 biologically independent samples and are presented as mean ± SEM. Statistical analysis was performed using a two-tailed unpaired *t*-test. Source data are provided as a Source Data file.

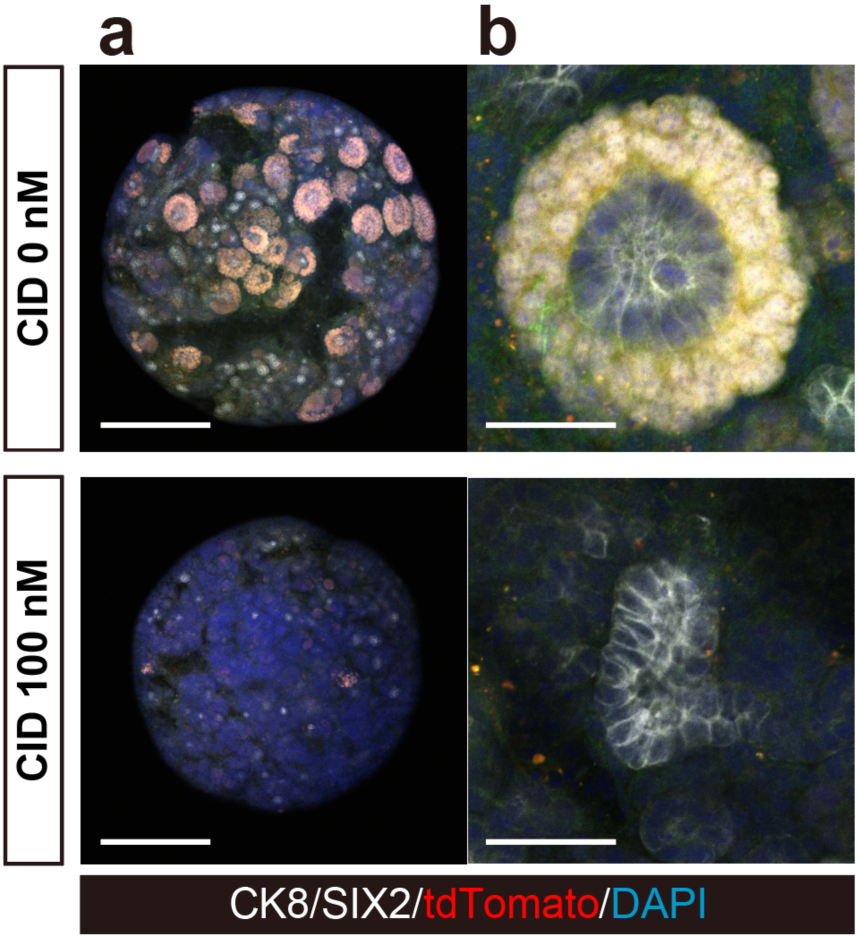

Supplementary Fig. 10 | Cell ablation induction in dissociated and re-aggregated fetal Six2-iC9$^{+/+}$ kidney cell spheres.

a Whole-mount immunostaining images of fetal Six2-iC9$^{+/+}$ kidney cell spheres cultured for 4 days without CID or with 100 nM CID added from the point of re-aggregation, showing SIX2^{+}/tdTomato^{+} NPCs surrounding CK8^{+} ureteric buds. Scale bars, 200 µm. **b** Magnified images of (**a**). Scale bars, 50 µm. CID, chemical inducer of dimerization; CK8, cytokeratin 8.

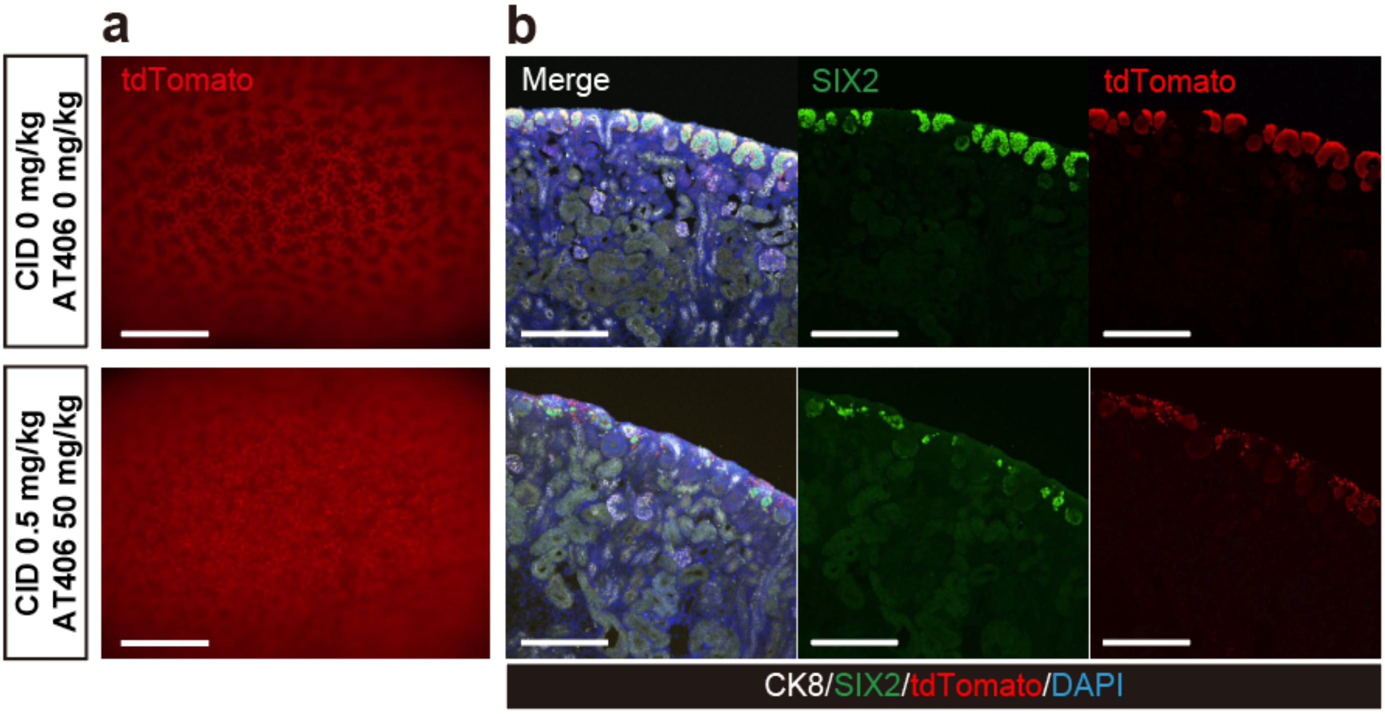

Supplementary Fig. 11 | NPC ablation in heterozygous neonatal kidneys by co-administration of XIAP inhibitor and CID.

a Fluorescence stereomicroscopic images of P2.5 neonatal Six2-iC9$^{+/-}$ kidneys without drug administration or 1 day after subcutaneous administration of 0.5 mg/kg CID and 50 mg/kg AT406 on P1.5. Scale bars, 500 µm. **b** Frozen section immunostaining images of (**a**), showing SIX2^{+}/tdTomato^{+} NPCs. Scale bars, 200 µm. CID, chemical inducer of dimerization; CK8, cytokeratin 8.

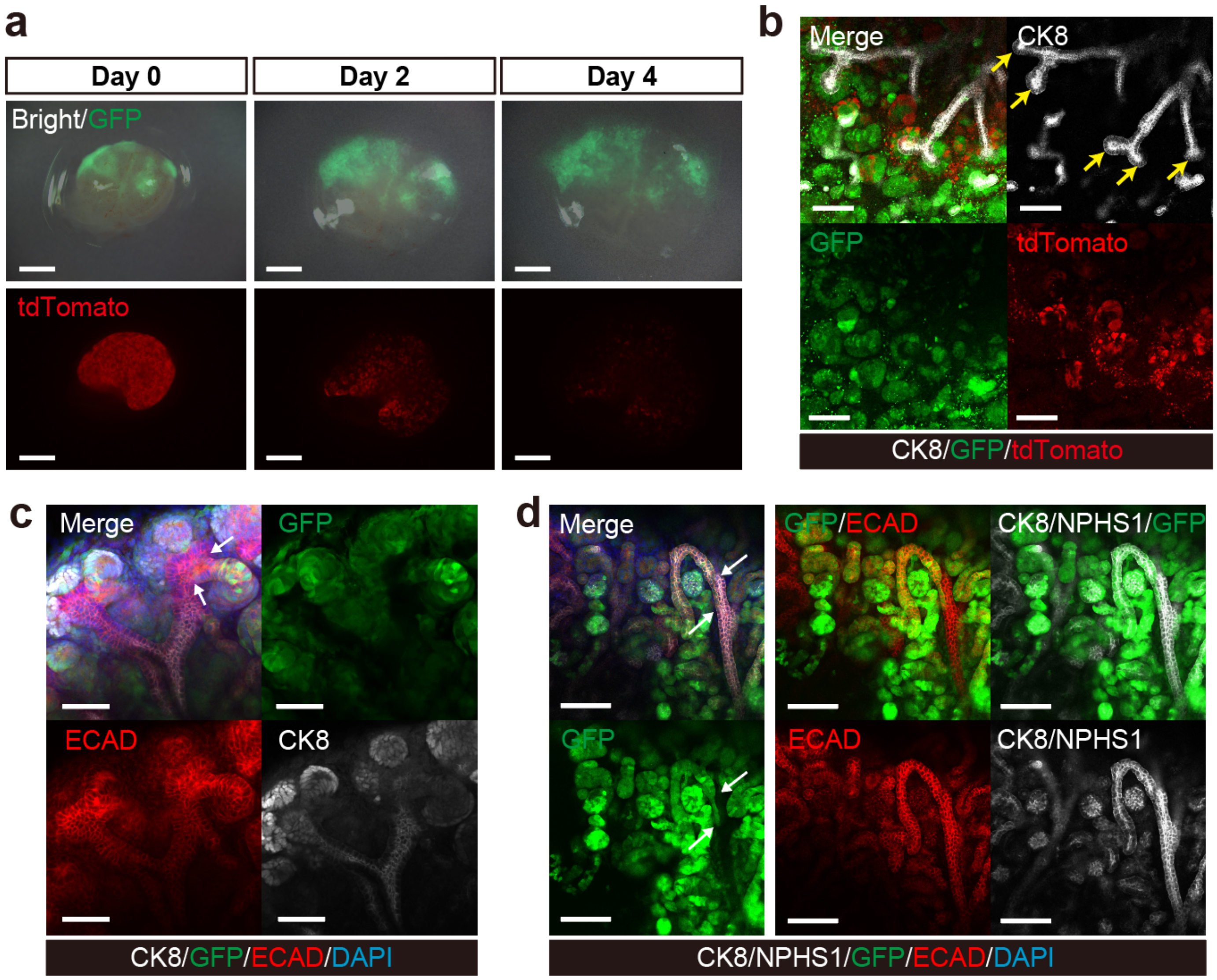

Supplementary Fig. 12 | Replacement and maturation of nephron progenitor cells using fetal Six2-iC9 kidneys as a scaffold.

a Fluorescence stereomicroscopic images of fetal Six2-iC9$^{+/+}$ kidneys with rat RPC injection, cultured with 100 nM CID for 4 days on the air–liquid interface, showing tdTomato^{+} host NPCs and GFP^{+} donor RPCs. Scale bars, 500 μm. **b–d** Whole-mount immunostaining images of Six2-iC9$^{+/-}$ kidneys with mouse RPC injection cultured with 100 nM CID and 10 μM AT406 for 4 days (**b, c**) and 7 days (**d**). Yellow arrows in (**b**) represent branching CK8^{+}/GFP^{-} host ureteric bud tips surrounded by GFP^{+} donor NPCs. White arrows in (**c, d**) indicate the connecting points between ECAD^{+}/GFP^{+} donor distal tubules and CK8^{+}/GFP^{-} host collecting ducts. Scale bars, 100 μm. CK8, cytokeratin 8; ECAD, E-cadherin; GFP, green fluorescent protein.

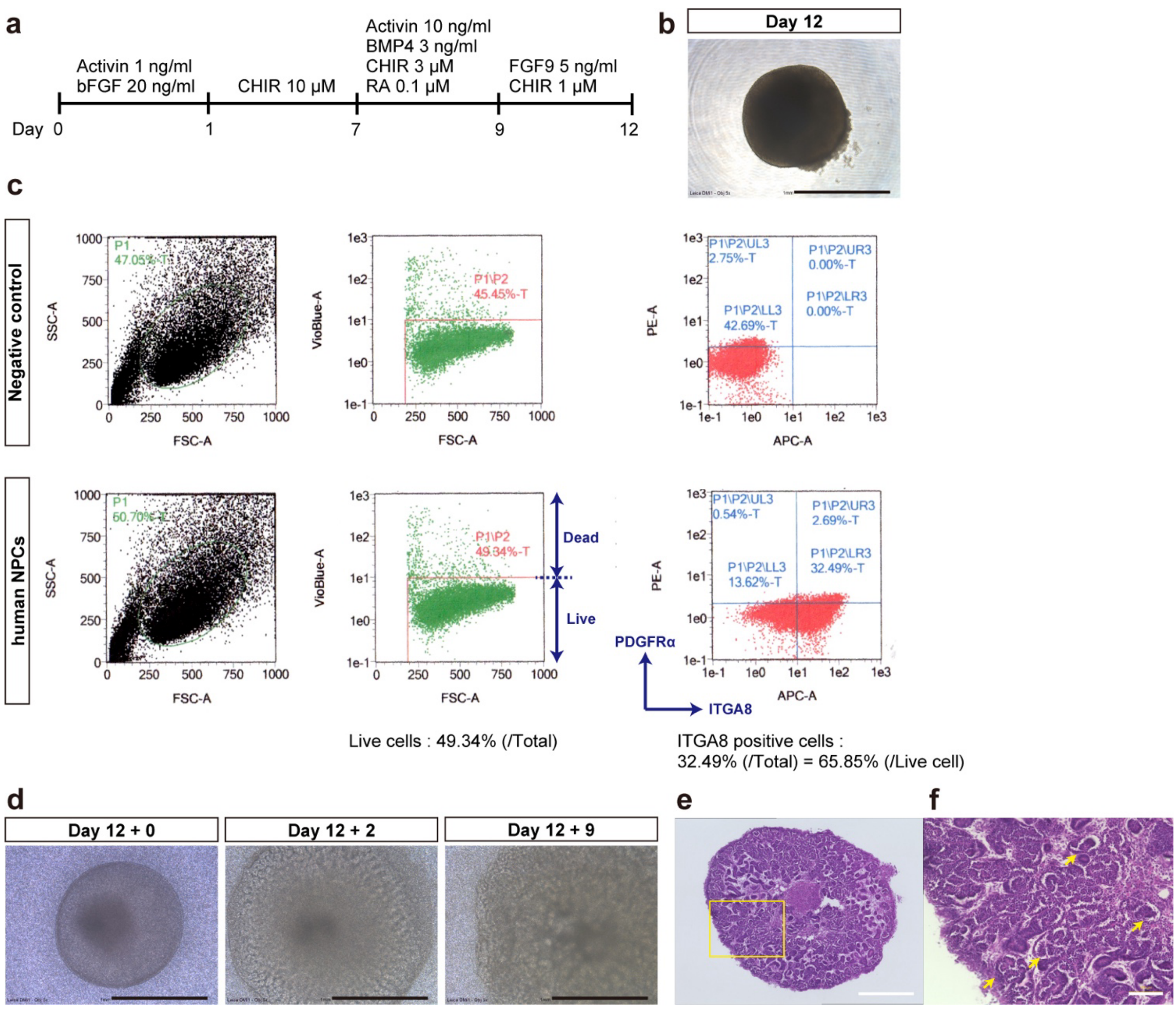

Supplementary Fig. 13 | Purity and differentiation capacity of induced human NPCs.

a A schematic illustrating the protocol for inducing nephron progenitor cells (NPCs) from human induced pluripotent stem cells (hiPSCs). **b** Microscopic images of NPC spheres on day 12. Scale bars, 1 mm. **c** Representative images of the flow cytometric analysis of NPC spheres. **d** Microscopic images of human NPC spheres cultured on the air–liquid interface for 9 days. Scale bars, 1 mm. **e** Hematoxylin and eosin-stained section of (**c**). Scale bars, 500 μm. **f** A magnified image of the yellow square of (**e**). Yellow arrows indicate glomeruli. Scale bars, 100 μm. ITGA8, integrin subunit alpha 8; PDGFRα, platelet-derived growth factor receptor alpha.

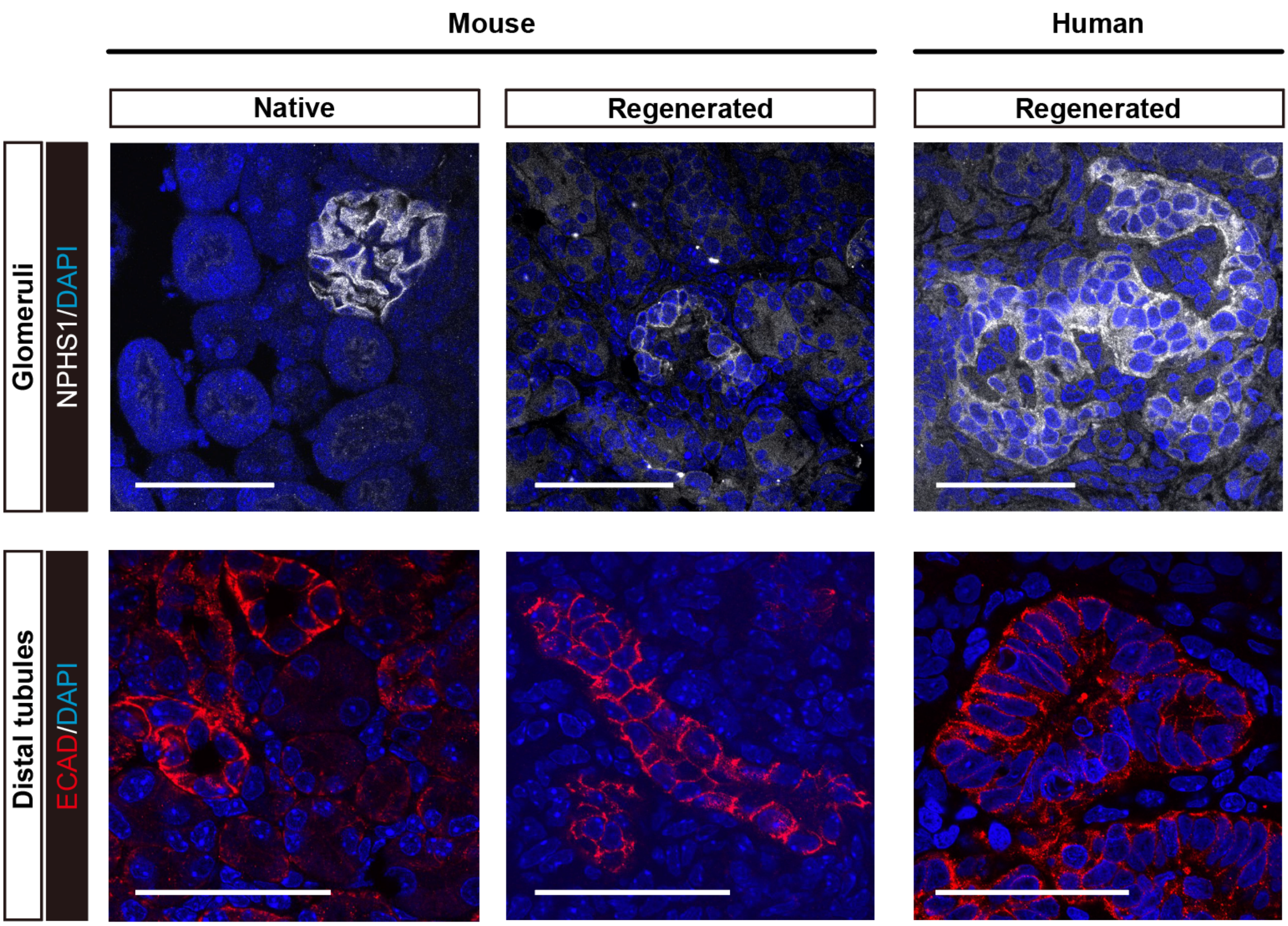

Supplementary Fig. 14 | Comparison of the size of regenerated nephrons between humans and mice.

Glomeruli (NPHS1$^+$) and distal tubules (ECAD$^+$) of (1) native kidneys harvested from 2-month-old mice (left), (2) *in vivo* regenerated mouse nephrons in fetal wild-type mouse kidneys collected after 7 days following transplantation into NOG mice (middle), and (3) *in vivo* regenerated human nephrons in fetal Six2-iC9$^{+/+}$ kidneys, following the injection of human NPCs, which were cultured for 3 days, transplanted into NOG mice, and collected after 7 days (right). Scale bars, 50 μm. ECAD, E-cadherin.

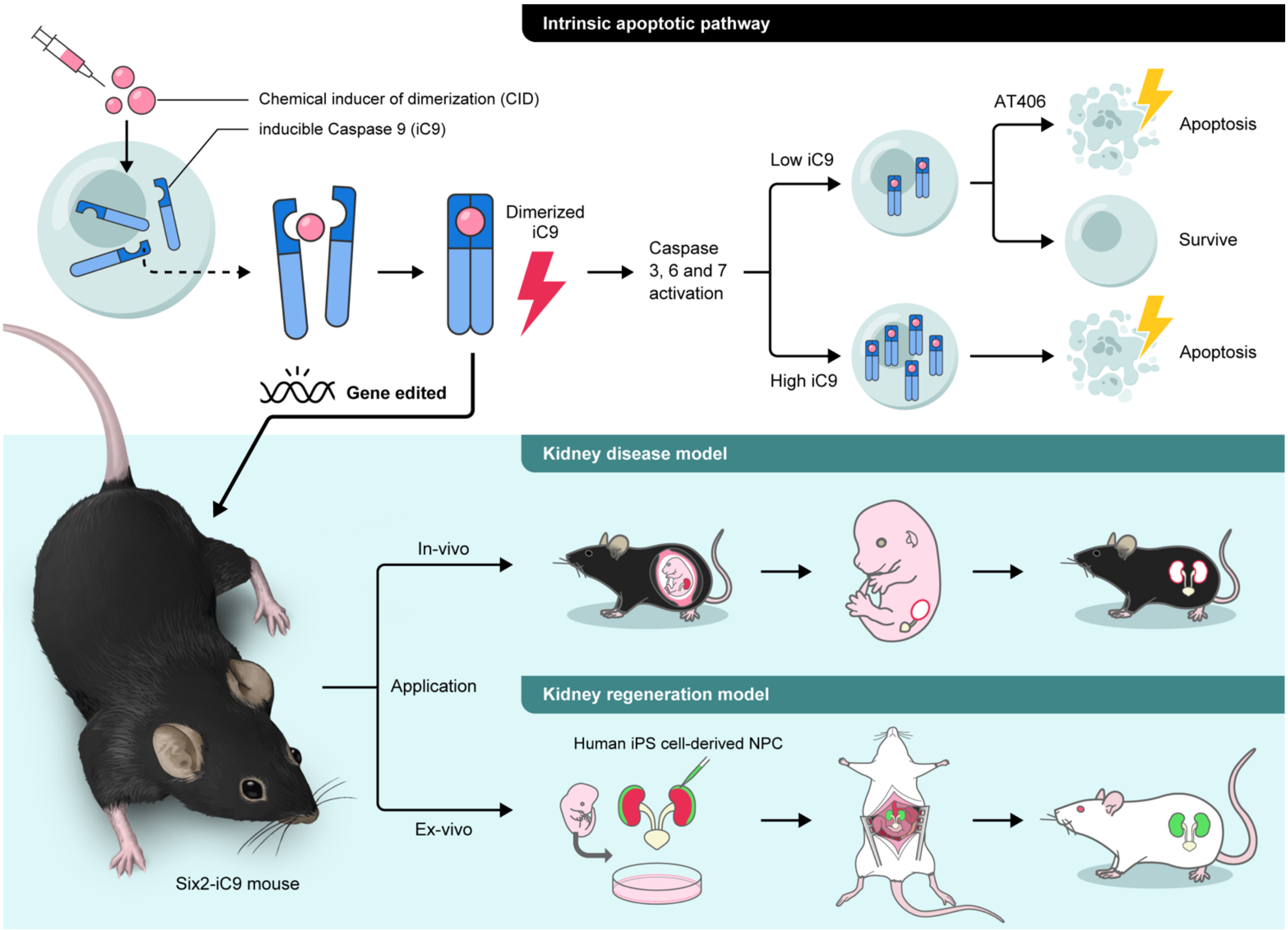

Supplementary Fig. 15 | A schematic illustrating this study.

The figure illustrates the inducible Caspase 9 (iC9)-mediated apoptosis system, where chemical inducer of dimerization (CID) induces iC9 dimerization, activating the intrinsic apoptotic pathway and enabling targeted cell ablation. High iC9 expression is required to induce apoptosis; however, apoptosis can also be triggered with low iC9 expression through the co-administration of AT406, an inhibitor of X-linked inhibitor of apoptosis protein. The Six2-iC9 mouse model enables controlled apoptosis in nephron progenitor cells (NPCs), allowing various applications: disease modeling with adjustable nephron loss, ranging from congenital kidney deficiency to severe chronic kidney disease in viable animals, and a scaffold for human–mouse chimeric kidney regeneration, using human induced pluripotent stem cells (iPSCs)-derived NPCs. *This figure was created by LAIMAN, Inc.*

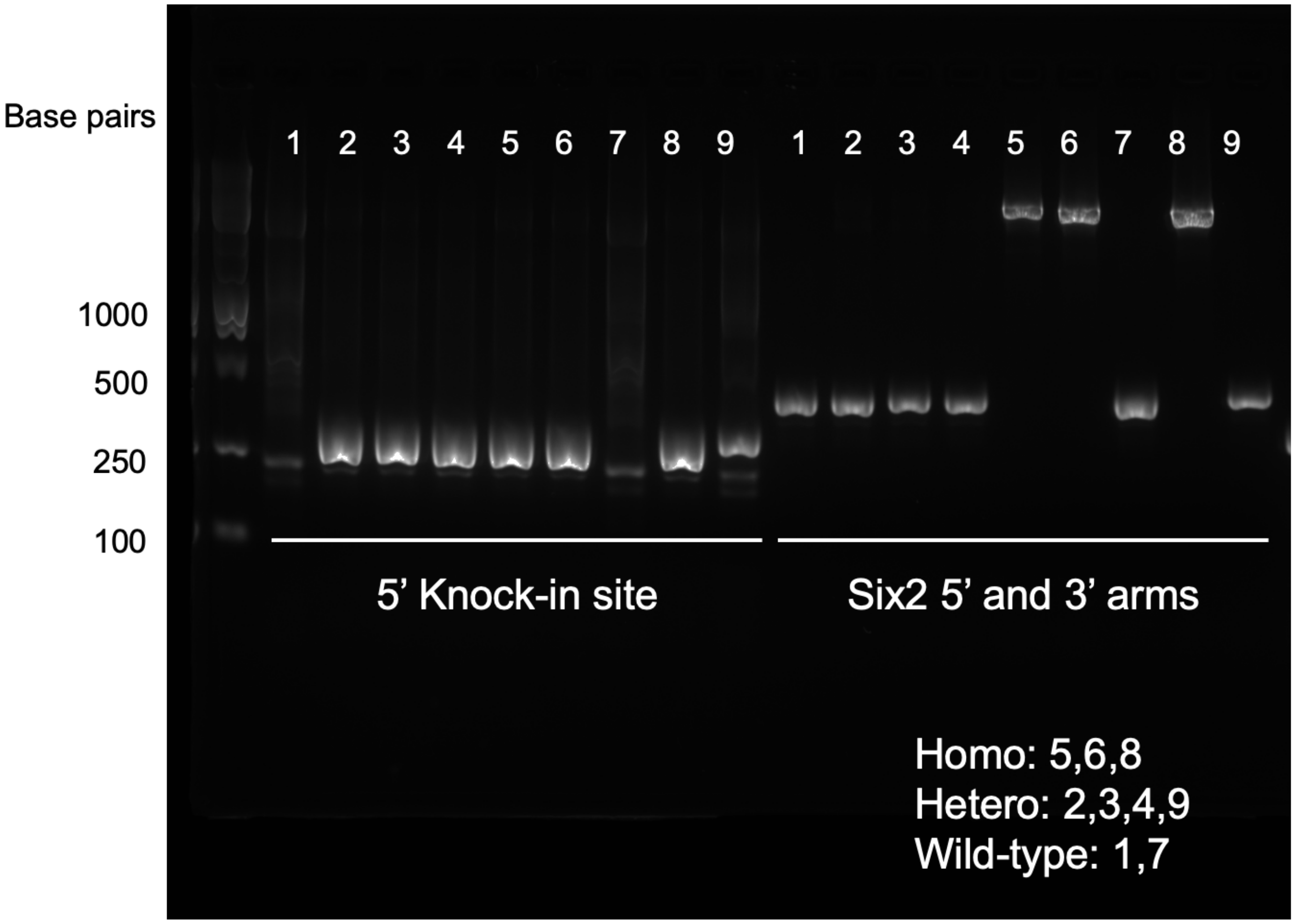

Supplementary Fig. 16 | An uncropped scan of all blots in Supplementary Fig. 1c.

Supplementary Table 1 | The primer sequences for genomic PCR.

Primers	Primer sequences	Products		
		Six2-iC9$^{+/+}$	Six2-iC9$^{+/-}$	Wild type
Six2 5' and 3' arms	Forward: gcagttccgaggatgagaag<br>Reverse: caaaggataccgagcagacc	3200 bp	420 bp	420 bp
5' knock-in site	Forward: tgcagcatcaccacagcctgc<br>Reverse: ctcggatcacctcctgcttg	318 bp	318 bp	null

Supplementary Table 2 | List of primary antibodies for immunostaining.

Antibodies	Source	Identifier	Host	Dilution
CK8 (TROMA-I)	DSHB	N/A	Rat	1:100
SIX2	Proteintech	Cat. # 11562-1-AP	Rabbit	1:100
tdTomato	MyBioSource	Cat. # MBS448122	Goat	1:100
NPHS1	Progen	Cat. # GP-N2	Guinea pig	1:100
ECAD	Cell Signaling Technology	Cat. # 3195S	Rabbit	1:100
IBA1	Novus Biologicals	Cat. # NB100-1028	Goat	1:100
GFP	Abcam	Cat. # ab13970	Chicken	1:200
LTL	Vector	Cat. # B-1325	Biotinylated	1:200
TFAP2B	Proteintech	Cat. #13183-1-AP	Rabbit	1:100
CD31	R&D systems	Cat. # AF3628	Goat	1:100

CK8, cytokeratin 8; ECAD, E-cadherin; GFP, green fluorescent protein; IBA1, Ionized calcium binding adaptor molecule 1; LTL, lotus tetragonolobus lectin; TFAP2B, transcription factor AP-2 beta.

Supplementary Table 3 | List of primary antibodies for capillary Western immunoassay.

Antibodies	Source	Identifier	Host	Dilution	Target size (kDa)
tdTomato	Cell Signaling Technology	Cat. # 20163S	Rabbit	1:10	60
β-actin	Cell Signaling Technology	Cat. # 3700S	Mouse	1:50	49
SIX2	Proteintech	Cat. # 11562-1-AP	Rabbit	1:50	44

Supplementary Table 4 | List of TaqMan Gene Expression Assays for RT-qPCR.

Gene Name	Source	Identifier
Gapdh	Thermo Fisher Scientific	Cat. # Mm99999915_g1
Fn1	Thermo Fisher Scientific	Cat. # Mm01256744_m1
Acta2	Thermo Fisher Scientific	Cat. # Mm00725412_s1
Col1a1	Thermo Fisher Scientific	Cat. # Mm00801666_g1

Acta2, actin alpha 2, smooth muscle; *Col1a1*, collagen type I alpha 1 chain; *Fn1*, fibronectin 1.
