# Supplementary material for: Caspase 9-induced apoptosis enables efficient fetal cell ablation and disease modeling
Source: Nat Commun. 2025 Mar 15;16:2572. doi: 10.1038/s41467-025-57795-6 (PMC11910536; doi:10.1038/s41467-025-57795-6)
Supplement: Supplementary file 2 — Description of Additional Supplementary Files [file 41467_2025_57795_MOESM2_ESM.pdf]

Description of Additional Supplementary Files

File Name: Supplementary Movie 1
Description: Immunostaining showing intraspecies replacement of nephron progenitor cells using rat fetal kidney-derived cells (GFP+) in a fetal Six2-iC9+/+ kidney. White, cytokeratin 8 and SIX2; red, tdTomato; green, GFP; blue, DAPI.

File Name: Supplementary Movie 2
Description: Immunostaining showing engraftment of rat nephron progenitor cells (SIX2+ , GFP+) replacing host nephron progenitor cells (SIX2+ , tdTomato+) in a fetal Six2-iC9+/+ kidney. White, cytokeratin 8 and Six2; red, tdTomato; green, GFP; blue, DAPI.

File Name: Supplementary Movie 3
Description: Immunostaining showing partial infiltration of human nephron progenitor cells (SIX2+ , GFP+) into the mouse cap mesenchyme without ablation of host nephron progenitor cells (SIX2+ , tdTomato+). White, cytokeratin 8 and SIX2; red, tdTomato; green, GFP; blue, DAPI.

File Name: Supplementary Movie 4
Description: Immunostaining showing engraftment of human nephron progenitor cells (SIX2+ , GFP+) replacing host nephron progenitor cells (SIX2+ , tdTomato+) in a fetal Six2-iC9+/+ kidney. White, cytokeratin 8 and SIX2; red, tdTomato; green, GFP; blue, DAPI.

File Name: Supplementary Movie 5
Description: Immunostaining showing in vivo differentiation of human nephron progenitor cells into distal tubules (E-cadherin+, cytokeratin 8− , GFP+) connected to a mouse collecting duct (E-cadherin+ , cytokeratin 8+ , GFP−). White, cytokeratin 8; red, E-cadherin; green, GFP; blue, DAPI.
