# Supplementary material for: Caspase 9-induced apoptosis enables efficient fetal cell ablation and disease modeling
Source: Nat Commun. 2025 Mar 15;16:2572. doi: 10.1038/s41467-025-57795-6 (PMC11910536; doi:10.1038/s41467-025-57795-6)
Supplement: Supplementary file 8 — Reporting Summary [file 41467_2025_57795_MOESM8_ESM.pdf]

nature portfolio

Corresponding author(s): Shuichiro Yamanaka

Last updated by author(s): January 29, 2025

Reporting Summary

Nature Portfolio wishes to improve the reproducibility of the work that we publish. This form provides structure for consistency and transparency in reporting. For further information on Nature Portfolio policies, see our Editorial Policies and the Editorial Policy Checklist.

Please do not complete any field with "not applicable" or n/a. Refer to the help text for what text to use if an item is not relevant to your study.
For final submission: please carefully check your responses for accuracy; you will not be able to make changes later.

Statistics

For all statistical analyses, confirm that the following items are present in the figure legend, table legend, main text, or Methods section.

n/a	Confirmed	
☐	☒	The exact sample size (*n*) for each experimental group/condition, given as a discrete number and unit of measurement
☐	☒	A statement on whether measurements were taken from distinct samples or whether the same sample was measured repeatedly
☐	☒	The statistical test(s) used AND whether they are one- or two-sided<br>*Only common tests should be described solely by name; describe more complex techniques in the Methods section.*
☐	☒	A description of all covariates tested
☒	☐	A description of any assumptions or corrections, such as tests of normality and adjustment for multiple comparisons
☐	☒	A full description of the statistical parameters including central tendency (e.g. means) or other basic estimates (e.g. regression coefficient) AND variation (e.g. standard deviation) or associated estimates of uncertainty (e.g. confidence intervals)
☐	☒	For null hypothesis testing, the test statistic (e.g. *F*, *t*, *r*) with confidence intervals, effect sizes, degrees of freedom and *P* value noted<br>*Give P values as exact values whenever suitable.*
☒	☐	For Bayesian analysis, information on the choice of priors and Markov chain Monte Carlo settings
☒	☐	For hierarchical and complex designs, identification of the appropriate level for tests and full reporting of outcomes
☒	☐	Estimates of effect sizes (e.g. Cohen's *d*, Pearson's *r*), indicating how they were calculated

Our web collection on statistics for biologists contains articles on many of the points above.

Software and code

Policy information about availability of computer code

Data collection: LSM880; BZ-X800; MACSQuant Analyzer & MACSQuantify Software; Wes

Data analysis: Graph Pad Prism version 8.4.3; MACSQuant Analyzer & MACSQuantify Software; Image J version 2.0.0 , Compass for Simple Western version 6.1.0

For manuscripts utilizing custom algorithms or software that are central to the research but not yet described in published literature, software must be made available to editors and reviewers. We strongly encourage code deposition in a community repository (e.g. GitHub). See the Nature Portfolio guidelines for submitting code & software for further information.

Data

Policy information about availability of data

All manuscripts must include a data availability statement. This statement should provide the following information, where applicable:

- Accession codes, unique identifiers, or web links for publicly available datasets
- A description of any restrictions on data availability
- For clinical datasets or third party data, please ensure that the statement adheres to our policy

All sequencing data generated in this study have been deposited in Gene Expression Omnibus (GEO) with the GEO accession number GSE282139 (https://www.ncbi.nlm.nih.gov/geo/query/acc.cgi?acc=GSE282139). The raw data used in all figures in this study are provided in the Source Data.

Research involving human participants, their data, or biological material

Policy information about studies with human participants or human data. See also policy information about sex, gender (identity/presentation), and sexual orientation and race, ethnicity and racism.

Reporting on sex and gender	N/A
Reporting on race, ethnicity, or other socially relevant groupings	N/A
Population characteristics	N/A
Recruitment	N/A
Ethics oversight	N/A

Note that full information on the approval of the study protocol must also be provided in the manuscript.

Field-specific reporting

Please select the one below that is the best fit for your research. If you are not sure, read the appropriate sections before making your selection.

☒ Life sciences ☐ Behavioural & social sciences ☐ Ecological, evolutionary & environmental sciences

For a reference copy of the document with all sections, see nature.com/documents/nr-reporting-summary-flat.pdf

Life sciences study design

All studies must disclose on these points even when the disclosure is negative.

Sample size	No sample size calculation was performed. For statistical analyses, quantitative evaluations in immunostaining were performed on randomly selected sections from at least three biologically independent samples. Quantitative evaluations in Wes were performed on four biologically independent samples. Quantative evaluations of serun and urinary analyses were performed on at least eight individuals, while quantitative evaluations in qPCR were performed on eight individuals.
Data exclusions	No data were excluded.
Replication	All experiments were independently repeated at least three times with similar results, and representative fluorescence stereomicroscopy and immunostaining images are shown in the figures.
Randomization	The administration of CID and AT406 was randomly assigned to fetal kidneys, without consideration for their sex.
Blinding	Immunostaining counts were conducted by two experimenters who were blinded to the experimental conditions. Blood tests were conducted by an experimenter who was blinded to the experimental conditions. Urine evaluation was conducted in SRL that was blinded to the experimental conditions.

Behavioural & social sciences study design

All studies must disclose on these points even when the disclosure is negative.

Study description	N/A
Research sample	N/A
Sampling strategy	N/A
Data collection	N/A
Timing	N/A
Data exclusions	N/A
Non-participation	N/A
Randomization	N/A

Ecological, evolutionary & environmental sciences study design

All studies must disclose on these points even when the disclosure is negative.

Study description	N/A
Research sample	N/A
Sampling strategy	N/A
Data collection	N/A
Timing and spatial scale	N/A
Data exclusions	N/A
Reproducibility	N/A
Randomization	N/A
Blinding	N/A

Did the study involve field work? ☐ Yes ☒ No

Field work, collection and transport

Field conditions	N/A
Location	N/A
Access & import/export	N/A
Disturbance	N/A

Reporting for specific materials, systems and methods

We require information from authors about some types of materials, experimental systems and methods used in many studies. Here, indicate whether each material, system or method listed is relevant to your study. If you are not sure if a list item applies to your research, read the appropriate section before selecting a response.

Materials & experimental systems

n/a	Involved in the study	
☐	☒	Antibodies
☐	☒	Eukaryotic cell lines
☒	☐	Palaeontology and archaeology
☐	☒	Animals and other organisms
☒	☐	Clinical data
☒	☐	Dual use research of concern
☒	☐	Plants

Methods

n/a	Involved in the study	
☒	☐	ChIP-seq
☐	☒	Flow cytometry
☒	☐	MRI-based neuroimaging

Antibodies

Antibodies used	In Flow cytometry, biotinylated anti-ITGA8 (R&D Systems), allophycocyanin-conjugated streptavidin (BioLegend), and phycoerythrin-conjugated anti-PDGFRA (BioLegend) were used for cell staining. Antibodies used in immunostaining and capillary Western immunoassay are listed in Supplementary Tables 2 and 3.
Validation	All antibodies used are commercially available and have been validated by the manufacturer. In all immunostaining experments, negative controls without first antibodies were prepared.

Eukaryotic cell lines

Policy information about cell lines and Sex and Gender in Research

Cell line source(s)	EGFP-labeled iPSC line (317-12-Ff). Established from dermal fibroblast, Caucasian, female, 36-year-old.
Authentication	Fabian Oceguera-Yanez, et al. Methods. 15;101:43-55.(2016)
Mycoplasma contamination	The cell line was tested negative for mycoplasma contamination.
Commonly misidentified lines (See ICLAC register)	No commonly misidentified cell lines were used in the study

Palaeontology and Archaeology

Specimen provenance	
Specimen deposition	
Dating methods	

☐ Tick this box to confirm that the raw and calibrated dates are available in the paper or in Supplementary Information.

Ethics oversight	

Note that full information on the approval of the study protocol must also be provided in the manuscript.

Animals and other research organisms

Policy information about studies involving animals; ARRIVE guidelines recommended for reporting animal research, and Sex and Gender in Research

Laboratory animals	Six2-iC9 mice, ROSA26-iC9 mice, and Six2-iC9 rats were established in this study. Pregnant female B6 mice, EGFP mice, EGFP rats, adult male and female B6 mice, and adult female ICR mice, were purchased from SLC. Adult male NOG mice were purchased from CLEA. Six2-EGFP/CreERT2 mice and Six2-EGFP/Cre mice were purchased from Jackson Laboratory.
Wild animals	This study did not involve any wild animals.
Reporting on sex	Both male and female fetal and neonatal mice and rats were used. The sex was distinguished in Fig. 2a,d,g,h,j,k, and 5i .
Field-collected samples	All animals were maintained on a 12-hour light/dark cycle with free access to standard diet and water.
Ethics oversight	All animal experiments were performed following the Guidelines for the Proper Conduct of Animal Experiments of the Science Council of Japan (2006) and were approved by the Institutional Animal Care and Use Committee of the Jikei University School of Medicine (approval numbers: 2020-039 and D2021-007).

Note that full information on the approval of the study protocol must also be provided in the manuscript.

Clinical data

Policy information about clinical studies

All manuscripts should comply with the ICMJE guidelines for publication of clinical research and a completed CONSORT checklist must be included with all submissions.

Clinical trial registration	
Study protocol	
Data collection	
Outcomes	

Dual use research of concern

Policy information about dual use research of concern

Hazards

Could the accidental, deliberate or reckless misuse of agents or technologies generated in the work, or the application of information presented in the manuscript, pose a threat to:

No | Yes
- [ ] [ ] Public health
- [ ] [ ] National security
- [ ] [ ] Crops and/or livestock
- [ ] [ ] Ecosystems
- [ ] [ ] Any other significant area

Experiments of concern

Does the work involve any of these experiments of concern:

No | Yes
- [ ] [ ] Demonstrate how to render a vaccine ineffective
- [ ] [ ] Confer resistance to therapeutically useful antibiotics or antiviral agents
- [ ] [ ] Enhance the virulence of a pathogen or render a nonpathogen virulent
- [ ] [ ] Increase transmissibility of a pathogen
- [ ] [ ] Alter the host range of a pathogen
- [ ] [ ] Enable evasion of diagnostic/detection modalities
- [ ] [ ] Enable the weaponization of a biological agent or toxin
- [ ] [ ] Any other potentially harmful combination of experiments and agents

Plants

Seed stocks

Novel plant genotypes

Authentication

ChIP-seq

Data deposition

- [ ] Confirm that both raw and final processed data have been deposited in a public database such as GEO.
- [ ] Confirm that you have deposited or provided access to graph files (e.g. BED files) for the called peaks.

Data access links
May remain private before publication.

Files in database submission

Genome browser session
(e.g. UCSC)

Methodology

Replicates

Sequencing depth

Antibodies

Peak calling parameters

Data quality

Software

X

Flow Cytometry

Plots

Confirm that:

☒ The axis labels state the marker and fluorochrome used (e.g. CD4-FITC).

☒ The axis scales are clearly visible. Include numbers along axes only for bottom left plot of group (a 'group' is an analysis of identical markers).

☒ All plots are contour plots with outliers or pseudocolor plots.

☒ A numerical value for number of cells or percentage (with statistics) is provided.

Methodology

Sample preparation	To evaluate the percentage of NPCs from each induction, flow cytometry was performed. Biotinylated anti-ITGA8, allophycocyanin-conjugated streptavidin, and phycoerythrin-conjugated anti-PDGFRA were used for cell staining.
Instrument	MACSQuant Analyzer & MACSQuantify Software
Software	MACSQuant Analyzer & MACSQuantify Software
Cell population abundance	A minimum of 5 x 10^5 cells was secured in each experiment.
Gating strategy	The APC (Itga8)-positive and PE (PDGFRA)-negative fraction was designated as the NPC population. The cut-offs for APC and PE fluorescence were determined using a negative control without the primary antibodies.

☒ Tick this box to confirm that a figure exemplifying the gating strategy is provided in the Supplementary Information.

Magnetic resonance imaging

Experimental design

Design type	
Design specifications	
Behavioral performance measures	
Imaging type(s)	
Field strength	
Sequence & imaging parameters	
Area of acquisition	

Diffusion MRI ☐ Used ☐ Not used

Preprocessing

Preprocessing software	
Normalization	
Normalization template	
Noise and artifact removal	
Volume censoring	

Statistical modeling & inference

Model type and settings	
Effect(s) tested	

Specify type of analysis: ☐ Whole brain ☐ ROI-based ☐ Both

Statistic type for inference

(See Eklund et al. 2016)

Correction

Models & analysis

n/a	Involved in the study
☐	☐ Functional and/or effective connectivity
☐	☐ Graph analysis
☐	☐ Multivariate modeling or predictive analysis

Functional and/or effective connectivity

Graph analysis

Multivariate modeling and predictive analysis

This checklist template is licensed under a Creative Commons Attribution 4.0 International License, which permits use, sharing, adaptation, distribution and reproduction in any medium or format, as long as you give appropriate credit to the original author(s) and the source, provide a link to the Creative Commons license, and indicate if changes were made. The images or other third party material in this article are included in the article's Creative Commons license, unless indicated otherwise in a credit line to the material. If material is not included in the article's Creative Commons license and your intended use is not permitted by statutory regulation or exceeds the permitted use, you will need to obtain permission directly from the copyright holder. To view a copy of this license, visit http://creativecommons.org/licenses/by/4.0/
